# The State Board of Health

**Published:** 1875-06

**Authors:** 


*The State Board of Health.*

As our Georgia readers are aware, the Legislature at its late session passed a law creating the board above mentioned. We regard the law a good one for both the people and profession of the State, one which, in some form, has been sadly needed in the Empire State of the South.

The members of the board under the terms of the law, and by appointment by the Governor, are as follows: J. G. Thomas, M.D., Benj. M. Cromwell, M. D., H. F. Campbell, M. D., Geo. F. Cooper, M.D., C. B. Nottingham, M.D., H. H. Carleton, M.D., F. A. Stanford, M. D., J. P. Logan, M. D., G. W. Holmes, M. D., Hon. N. J. Hammond, Attorney General, Hon. W. L. Goldsmith, Comptroller General, and Professor G. S. Little, State Geologist.

The board met on the 9th inst., in this city, and organized by electing J. G. Thomas, M.D., of Savannah, President, and V. H. Taliaferro, M.D., of Atlanta, Secretary. The President then appointed the following committees:

*Finance.*—Logan, Carleton, Goldsmith.

*Stationery and Printing.*—Goldsmith, Logan, Little.

*Legislation.* — Hammond, Campbell, Nottingham, Stanford, Holmes.

*Prudential.*—Logan, Goldsmith, Hammond.

*Endemic, Epidemic and Contagious Diseases.*—Campbell, Nottingham, Cromwell.

*Hygiene of Schools, Prisons and Public Institutions.*—Nottingham, Stanford, Cooper.

The board moves off well. The officers selected are good men and will achieve success for the law, if success is possible. The members work for no remuneration. We trust, therefore, that each one in his proper district, will receive all the aid necessary from the profession, for the attainment of the objects contemplated.

In some future number of this journal we purpose giving the full text of the law for the benefit of the profession of the State.

